# Alpha-glucosidase inhibitory activities of astilbin contained in *Bauhinia strychnifolia* Craib. stems: an investigation by in silico and in vitro studies

**DOI:** 10.1186/s12906-023-03857-5

**Published:** 2023-01-30

**Authors:** Komgrit Eawsakul, Tassanee Ongtanasup, Ngamrayu Ngamdokmai, Kingkan Bunluepuech

**Affiliations:** 1 Department of Applied Thai Traditional Medicine, School of Medicine, Walailak University, Nakhon Si Thammarat, 80160 Thailand; 2 School of Allied Health Sciences and Research Excellence Center for Innovation and Health Products (RECIHP), Walailak University, Nakhon Si Thammarat, 80160 Thailand

**Keywords:** *Bauhinia strychnifolia* Craib., Astilbin, Alpha-glucosidase, Antihyperglycemic effect

## Abstract

**Introduction:**

Bioactive compounds from traditional medicines are good alternatives to standard diabetes therapies and may lead to new therapeutic discoveries. The stems of *Bauhinia strychnifolia* Craib. (BC) have a possible antihyperglycemic effect; However, the extraction of astilbin from BC has never been recorded in alpha-glucosidase inhibitory activities.

**Methods:**

Using liquid chromatography–mass spectrometry (LC–MS/MS), 32 compounds were detected in the BC extract. The screening was based on peak area. Seven compounds found. PASS recognized all seven compounds as potential alpha-glucosidase (AG) inhibitors. Astilbin and quercetin 3-rhamnoside were the most likely inhibitors of AG. Arguslab, AutoDock, and AutoDock Vina investigated the binding of the two compounds and AG. The binding stability was confirmed by molecular dynamics (MD). In addition, the optimum solvent extraction was studied via CosmoQuick, and extracts were examined with ^1^H-NMR prior to testing with AG.

**Results:**

All three software programs demonstrated that both compounds inhibit AG more effectively than acarbose. According to the sigma profile, THF is recommended for astilbin extraction. The BC extract with THF showed outstanding AG inhibitory action with an IC_50_ of 158 ± 1.30 µg mL-1, which was much lower than that of the positive control acarbose (IC_50_ = 190 ± 6.97 µg mL-1). In addition, astilbin from BC was found to inhibit AG strongly, IC50 = 22.51 ± 0.70 µg mL-1 through the extraction method of large-scale astilbin with THF has the best extraction capacity compared to other solvents, hence the initial stage of extraction employs THF to extract and precipitate them with ethyl acetate and water.

**Conclusion:**

*In silico* and *in vitro* studies reveal that astilbin inhibits AG and is superior to acarbose, validating its promise as an AG inhibitor. Overall, astilbin was the most bioactive component of BC for antidiabetic action.

## Introduction

Diabetes is the most common metabolic disorder worldwide, with various underlying causes contributing to hyperglycemia [1, 2]. Type 1 diabetes mellitus (T1DM) is defined by autoimmune damage to pancreatic beta cells that affects the functioning generation of insulin, which regulates blood glucose levels (five percent of all kinds of diabetes). In contrast, type 2 diabetes mellitus (T2DM) is caused by insulin resistance or insulin insufficiency (ninety percent of all kinds of diabetes). The remaining 5% comprises other subtypes [3]. The number of new instances continues to rise. The International Diabetes Federation forecasts that by 2045, there will be over 700 million cases of diabetes, up from 463 million in 2019 [4]. The scholarly community has created several chemical and synthetic antidiabetic medications throughout the years to aid in the management and prevention of diabetic complications [5]. Despite this, the deleterious effects of extended diabetic medication administration (such as digestive problems, obesity, and hypoglycemia) have shifted the focus of many individuals and researchers toward harmless alternatives [6]. Many diabetics have switched to medicinal plants since they are effective and safe and have few or no adverse effects [7].

A number of polyphenols, including isoflavones from soybeans, epicatechin, epicatechin gallate, catechin, epigallocatechin, tannic acid, saponins, chlorogenic acid, and glycyrrhizin from liquorice root, inhibit S-Glut-1-mediated glucose transfer from the gut. The transport of glucose from the stomach to the small intestine is slowed by saponins [8]. In diabetic rats, stilbene drugs such as resveratrol improve glucose homeostasis and insulin sensitivity [9]. It also ameliorates diabetic nephropathy, renal failure, and oxidative stress in diabetic rats. As resveratrol inhibits K + adenosine triphosphate (ATP) and K + (V) channel in beta cells, this is thought to be a plausible mechanism for insulin resistance delaying and insulin secretion reduction [10]. Quercetin, a polyphenolic molecule, protects against lipid peroxidation and oxidative stress, which aids antidiabetic action [11]. Quercetin's antidiabetic potential has been linked to its glucose uptake-inhibiting property and regulation of the mitogen-activated protein kinase pathway [12, 13].

*Bauhinia strychnifolia* Craib. (BC), commonly recognized as Yanang Dang, is among the promising herbs for treating T2DM*.* BC is a crimson-colored climbing shrub found mostly in northern Thailand. BC has a large proportion of phenols and flavonoids, which are believed to be beneficial for several biological functions, including antidiabetic effects [14]. Traditionally, the stems, roots, and leaves of BC were used in meals, health beverage products, health supplements, and Thai traditional medicine for vaccination, diet, relieving flu, minimizing alcohol problems, hay fever treatment, removing toxic substances, anti-inflammatory, anticancer, and antidiarrheal properties [15–17]. Multiple studies have established the pharmacological and biological effects of BC, which are also aligned with traditional Thai medicine and include antipyretic, hay fever relieving, detoxifying, antioxidant, antidiabetic, anticancer, antibacterial, and antiHIV properties [16, 18]. Only two investigations on antidiabetic activity have been published. In vitro, ethanolic extracts of BC inhibited AG, leading to decreased glucose [19] and cholesterol and triglyceride in the blood circulation of albino rats [20]. In addition, the two compounds 3,5,7,3′,5′-pentahydroxy-flavanonol-3-O-*α*-L-rhamnopyranoside and 3,5,7-trihydroxychromone-3-O-α-L-rhamnopyranoside that were extracted from the stems of BC with ethanol displayed potent inhibitory activity against cancer and AG [17, 19] However, further glucose-lowering actions of the stems of BC have not been explored, and the principal bioactive components remain unidentified. In this study, we examined the AG-inhibiting efficacy of BC and the bioactive components of BC. in the future, we expect that these results may promote the use of BC as a therapeutic alternative for T2DM or a source of important compounds in the development of the new drug.

## Materials and methods

### Materials

SwissADME, a free online application for ligand descriptors, was utilized in this study. The following system characteristics were used for the research. The computer simulation used in this research matches previous research [21]. The program defined these compatibility requirements for the aforementioned program. Thailand's Thail Oil Co., Ltd. supplied all solvents for the extraction and separation procedures. Germany's Sigma–Aldrich supplied para–nitrophenyl–*α*–D–glucopyranoside, AG from *Saccharomyces cerevisiae*, and acarbose. Labscan supplied the acetonitrile LC–MS grade (Bangkok, Thailand). A Millipore Milli-Q Integral 3 Water Purification System was used to create ultrapure water (Millipore, Bedford, MA, USA). Merck provided analytical-grade formic acid (Darmstadt, Germany).

### Plant materials

The *Bauhinia strychnifolia* Craib. Plant identification was performed at the Herbarium of the Department of Pharmacognosy and Pharmaceutical Botany, Faculty of Pharmaceutical Sciences, Prince of Songkla University by Dr. Kingkan Bunluepuech, Thai traditional medicine doctor. Its stems were gathered in 2020 in the Suan Ya Thai Thongnoppakhun herbal garden in Chonburi Province and voucher specimen was deposited with (SKP 072,021,901) voucher number. The collection of plant material complies with relevant guideline and regulations of the Plant Varieties Protection, Department of Agriculture, Ministry of Agriculture and Cooperatives, Thailand.

### Preparation of sample

The dried BC extracts were redissolved in methanol at a concentration of 10 mg/mL for chemical analysis. Plant experiments were performed in accordance with relevant guidelines and regulations.

### Analysis of chemical compounds by LC-ESI-QTOF-MS/MS

The chemical profile of BC was separated and elucidated using liquid chromatography (LC) and an electrospray ionization quadrupole time-of-flight mass spectrometer (LC-ESI-QTOF-MS/MS). The LC separation was performed using an Agilent 1260 Infinity Series HPLC System (Agilent Technologies, Waldbronn, Germany) and a Luna C-18 column, 5 µm, 4.6 mm × 150 mm. (Phenomenex Inc., Torrance, USA). The mobile phase was composed of a water phase (A) and acetonitrile phase (B), both of which contained 0.1% (v/v) formic acid to enhance ionization. The separation was a linear gradient condition of 5–95% A from 0–30 min, with a 10-min re-equilibration step between samples. The sample injection volume was 10 μL, and the flow rate of the solvent was 0.5 mL/min. Mass spectral data were acquired with a 6540 ultrahigh-definition Accurate Q-TOF-mass spectrometer (Agilent Technologies, Singapore) in the m/z range of 100–1000 Da, employing electrospray ionization (ESI) in both positive and negative ionization mode, and Agilent Mass Hunter Qualitative Analysis and Data Acquisition Software B.05.01 (Agilent Technologies, USA) were utilized for qualitative analysis.

### Probability of biological activity

Using the PASS web server (http://way2drug.com/PassOnline/), bioactivity prediction of BC compounds was performed. Categories of AG inhibitor activity were chosen for prediction. This program predicted the activity spectrum of a substance as probable activity (Pa) and probable inactivity (Pi). The prediction of the PASS spectrum is predicated on SAR assessment [22]. The PASS prediction findings were interpreted and used with versatility:

1. for a given molecule, only actions with Pa > Pi are deemed feasible;
2. if Pa is more than 0.70, the probability of finding the activity empirically is high;
3. if Pa is between 0.5 and 0.7, the probability of discovering the activity experimentally is reduced, and the molecule is likely not as comparable to recognized pharmacological drugs;
4. if Pa is less than 0.5, the probability of discovering the activity experimentally decreases, but the probability of discovering a structurally novel chemical increase.

### Molecular interaction and 2D/3D chemical modeling

Ligand-targeted enzyme dockings were developed to determine how effective a ligand was a more effective AG inhibitor (PDB 5ZCC) than acarbose, the typical diabetic treatment. The AG inhibitor in diabetes-treating BC has been explored. More than 30 chemicals were discovered from BC. The 3D structure and profiles of BC were retrieved from PubChem (https://pubchem.ncbi.nlm.nih.gov/) (Table 1). The initial geometry of all starting 3D molecular structure was optimized with UFF force field in Avogadro 1.2.0. Then, energy was minimized by Argus Lab. For AG, Polar hydrogens were added to the AG and Kollman charges were performed to determine their charge. The optimized ligands were docked using Argus Lab, AutoDock Vina, and AutoDock. Using Argus Lab, these chemicals were docked onto the AG target site. Docking effects were investigated when the levels of binding energy were lower than those of acarbose-AG. To determine the binding energies, the following parameters were used: the box dimensions were set to x = 66, y = 66, z = 94; the box location was set to x = 3.195, y = 48.279, z = 82.191; and the exhaustiveness was assigned to be 24. As for Autodock Vina, the grid spacing was set to 1 angstrom [23]. To confirm the binding affinity, AutoDock was employed by the 40-cubic-unit box whose x, y, and z coordinates were 3.549, 55.786, and 75.734, respectively. The chemical bindings, including hydrogen bonds and hydrophobic interactions, between the specified compounds and the AG pocket were visualized using Discovery Studio.

**Table 1:** The compound data of phytochemical BC for molecular docking

No	Compounds	Mw (g/mol)	Molecular Formula	PubChem ID
1	Acarbose	645.6	C_25_H_43_NO_18_	41774
2	Astilbin	450.4	C_21_H_22_O_11_	119258
3	Quercetin 3-rhamnoside	448.4	C_21_H_20_O_11_	5280459

### Evaluation of possible binding sites

The enzyme AG's crystal structure (PDB ID: 5ZCC) was derived from the RCSB Protein data library (PDB). By submitting the enzyme's PDB coordinates to the CavityPlus web service [24], the probable binding cavities for the enzyme's monomeric form were determined. Here, the unbound enzyme was utilized to determine the enzyme's possible binding sites. Additionally, the server identifies probable binding holes of the AG enzyme for every given ligand.

### Molecular dynamic simulation

Following the molecular docking, uncomplexed AG, astilbin-AG complex, and acarbose-AG complex were submitted to MD simulation. WEBGRO for macromolecular simulations (https://simlab.uams.edu/) (WebGRO for Macromolecular Simulations) was used to test the binding stability of the final complexes using Molecular Dynamics simulation. The whole system was solvated in water model, neutralized, and 0.15 M salt of NaCl was introduced using GROMOS96 43a1 force field settings. In 5000 steps, energy reduction was achieved using the steepest descent approach. Constant amount, volume, temperature (NVT/NPT) and pressure equilibration types were used. For a 1000 ps simulation duration and 1000 frames each simulation, the temperature was set to 300 K and the pressure was set to 1.0 bar. The requested simulation parameters were: Root Mean Square Deviation (RMSD) and intermolecular H-bonding (H-bonds). Topology file of the protein–ligand complexes were created using PRODRG sever [25].

### Solvent considerations

COSMOquick [26] was used to determine which solvents were good for extraction so that extraction yields of certain compounds could be raised. Six different solvents, including acetone, chloroform, methanol, ethanol, water, and tetrahydrofuran (THF), were used to test the chemicals. The process was performed as described in a previous study [21].

### Analysis of QSAR

Analysis of the quantitative structure–activity relationship (QSAR) and structure–activity relationship (SAR) rely on algorithms, and statistical techniques are widely used in many parts of pharmaceutical research, from inspection to optimization [27]. In this study, phytochemistry was subjected to QSAR assessment using EasyQSAR 1.0, which produced the following Eq. (1):

1 $$\mathrm{PredictedIC}50=\mathrm{Constant}+(\mathrm A1\mathrm B1)+(\mathrm A2\mathrm B2)+(\mathrm A3\mathrm B3)+...+(\mathrm{AnBn})$$

where each molecule's properties B_1_ through B_n_ are determined. The coefficients A_1_ through A_n_ are derived by fitting the parameter and biological activity changes. These B_1_, B_2_, B_3_, etc., are the descriptor variables of the QSAR equation [28]. For this objective, molecular descriptors were derived using the SwissADME tool descriptor, and the experimental values of the training sample were obtained from a previous publication [29]. The training dataset included three published inhibitor compounds and four ligand descriptors, namely, hydrogen donor (HD), LOGP, hydrogen acceptor (HA), and topological polar surface area (TPSA), which were utilized to forecast the QSAR model.

### Quantum compound computations

All investigated molecules were retrieved from PubChem and optimized by applying the universal force field (UFF) approach through the Avogadro software [30]. Afterwards, ORCA, a program for calculating the electronic structure, was utilized to conduct computations using B3LYP and quality basis sets def2-svp. Acarbose and astilbin structures were optimized in aqueous conditions to investigate the link between inhibitors' molecular characteristics and inhibitory efficiency [31]. IboView was utilized to view the electrical structures of molecules. The HOMO and LUMO energy gaps were used to compute the molecule chemical stability, as shown in Eq. (2).

2 $$\triangle\mathrm{EGap}=\mathrm{ELUMO}-\mathrm{EHOMO}$$

### Fractional isolation and NMR characterization

The stem powder of *B. strychnifolia* (9 kg) was extracted twice at room temperature with THF (56 L). Under negative pressure, the solvent was eliminated to provide a BC extract of 1.987 kg that was kept at 4 °C. According to the COSMOquick analysis, the polarity of astilbin was lower than that of the precipitate from the ethyl acetate:water fractions (98.0 g, 4.93% w/w). The ethyl acetate precipitate fraction:water (4 g) was isolated using 20% methanol in chloroform and chromatography on silica gel to make five fractions (F1-F5). Recrystallization of fraction F5 (316.4 mg) yielded astilbin (white crystal, 204 mg, 5.1% w/w). The chromatographic approach was used to extract astilbin, and the spectroscopic results were compared to those previously published [32].

### AG inhibition test

The THF extraction of BC and astilbin was dissolved in dimethyl sulfoxide (DMSO) at a concentration of 8 mg/mL. According to previously described tests [21], all extracts were found to inhibit AG. Briefly, the absorbance at 405 nm was used to find yellow p-nitrophenol, which was made from p-nitrophenyl-*α*-D-glucopyranoside (pNPG). The 8 mg/mL samples were generated by mixing 50 µL of sample solution with 50 µL of phosphate-buffered saline containing 2 mg/mL bovine serum albumin, 0.2 mg/mL sodium azide, and 50 µL of 1 unit/mL AG. DMSO (5%) and acarbose [33] were employed as negative and positive controls, respectively. All extracts were incubated for 2 min at 37 °C in an incubator with a controlled atmosphere. Then, each well received 4 mM pNPG. After 20 min, the reaction was stopped by adding 50 μL of 0.2 M Na_2_CO_3_. Six readings were taken using a microplate reader at 405 nm every 5 min to monitor the reaction. The % inhibition of AG was computed using Eq. (3). The results are expressed as a percentage showing 50% inhibition of AG activity (IC_50_).

3 $$\mathrm{AG}\;\mathrm{inhibition}\;(\%)=\left(\frac{{\mathrm{OD}}_{\mathrm{negative}\;\mathrm{control}}\;-{\mathrm{OD}}_{\mathrm{sample}}}{{\mathrm{OD}}_{\mathrm{negative}\;\mathrm{control}}}\right)\times\;100$$

### Statistical examination

All statistical analyses were performed using one-way ANOVA with SPSS 18. The samples were analyzed from six replicate measurements. When the *p* value was less than 0.05, statistical significance was established. Each value is represented by its mean and standard deviation (SD).

## Results and discussion

### LC–MS/MS analysis

This study employed LC–MS/MS to determine what compounds were present in BC extract. Considering retention time (RT), mass spectrometric findings from the data identification score, negative and positive electron spray ionization modes (ESI**-**/ESI +), and mass error, compounds with a relative abundance greater than 5% were considered for further investigation as AG inhibitors. Astilbin, neoastilbin, quercetin 3-rhamnoside, 5,7,3′,5′-tetrahydroxyflavanone, fisetin, quercitrin, and C16 spinganine are among the compounds mentioned in Tables 2 and 3 that account for more than 5% of BC compounds. A recent systematic review showed that 10 mg/kg quercetin can reduce the serum glucose concentration in mice [34]. It has been shown that quercetin from berry extract increases glucose absorption via a 5′ adenosine monophosphate-activated protein kinase (AMPK) route [35]. This happened despite the absence of insulin. Quercetin 3-rhamnoside, commonly known as quercitrin, might inhibit AG at 2.5 times lower levels than acarbose [29]. These results provide strong support for the notion that BC contains several antidiabetic substances that may assist with high blood sugar. However, the active components astilbin, fisetin, 5,7,3′,5′-tetrahydroxyflavanone, and C16 spinganine have not been found to reduce AG.

**Table 2:** Compounds identified in the THF BC extract with positive electrospray ionization (ESI +) MS analysis

S.No	RT	Name of the compound	Molecular Formulae	m/z	Peak area (%)
1	8.05	Methyl 6-*O*-galloyl-*β*-D-glucopyranoside	C_14_H_18_O_10_	345	0.57
2	9.27	Gentisic acid	C7H6O4	153	0.87
3	9.88	Ent-epicatechin-(2*α*- > 7,4*α*- > 8)-epicatechin 3-galactoside	C36H34O17	737	0.31
4	10.22	Hydroxyphenylethyl galloylrhamnoside	C21H24O10	471	1.09
5	10.31	ent-Epicatechin(4*α*- > 8)catechin	C30H26O12	577	1.08
6	10.50	Ent-epicatechin-(2*α*- > 7,4*α*- > 8)-epicatechin 3-galactoside	C36H34O17	737	0.41
7	10.65	Ent-epicatechin-(2*α*- > 7,4*α*- > 8)-epicatechin 3-galactoside	C36H34O17	737	0.30
8	11.02	3-*O*-*β*-D-Galactopyranosylproanthocyanidin A5'	C36H34O17	737	1.47
9	11.32	3-*O*-*β*-D-Galactopyranosylproanthocyanidin A5'	C36H34O17	737	1.19
10	11.71	3-*O*-*β*-D-Galactopyranosylproanthocyanidin A5'	C36H34O17	737	1.47
11	11.92	3,5,7-trihydroxychromone-3-*α*-L-rhamnopyranoside	C15H16O9	339	2.72
12	12.76	3,5,7,3',5'- pentahydroxyflavanonol-3-*O*-α-L-rhamnopyranoside	C21H22O11	449	4.77
13	12.98	Astilbin	C21H22O11	449	10.49
14	13.34	Neoastilbin	C21H22O11	449	14.10
15	13.50	Quercetin 3-rhamnoside	C21H20O11	447	14.10
16	13.85	5,7,3′,5′-Tetrahydroxyflavanone	C15H12O7	303	5.35
17	14.44	Eriodictin	C21H22O10	433	4.60
18	14.94	Cordeauxione	C14H12O7	291	2.41
19	15.54	Fustin	C15H12O6	287	1.87
20	16.85	Fisetin	C15H12O5	285	6.05
21	18.39	4,2',3',4'-Tetrahydroxychalcone	C15H12O5	271	1.04
22	18.64	Genistein	C15H10O5	269	1.57
23	19.07	5-Caffeoylshikimic acid	C16H16O8	335	2.31
24	19.52	Neobavachalcone	C17H14O5	297	1.84
25	19.723	4-coumaroylshikimate	C16H16O7	319	1.84
26	20.052	7,2',4'-Trihydroxyisoflavene	C15H12O4	255	0.66
27	20.273	5,8,12-trihydroxy-9-octadecenoic acid	C18H34O5	329	0.54
28	20.953	Kaempferol	C15H10O6	285	1.60
29	21.32	9,10,18-trihydroxy-12-octadecenoic acid	C18H34O5	329	3.00
30	21.732	Flavokawin B	C17H16O4	283	0.44
31	21.757	4,4'-Dihydroxy-2',6'-dimethoxychalcone	C17H16O5	299	0.92
32	24.131	2,4'-Dihydroxy-4-methoxydihydrochalcone	C16H16O4	271	0.98
33	24.485	Isoliquiritigenin 2'-methy ether	C16H14O4	269	0.56
34	26.176	*β*,2-Dihydroxy-4,6-dimethoxy-3-methylchalcone	C18H18O5	313	1.58
35	27.007	Dihydroxystearic acid	C18H36O4	315	1.07
36	27.869	Gingerglycolipid C	C33H60O14	715	0.47

**Table 3:** Compounds identified in the THF BC extract with negative electrospray ionization (ESI-) MS analysis

S.No	RT	Name of the compound	Molecular Formulae	m/z	Peak area (%)
1	2.626	2-Amino-3-methyl-1-butanol	C5H13NO	104	1.79
2	3.099	Methyl *β-*D-glucopyranoside	C7H14O6	195	8.98
3	3.51	6-(2-aminoethyl)benzene-1,2,3,4,5-pentol	C8H11NO5	202	2.91
4	9.063	2'',3''-Di-*O*-p-coumaroylafzelin	C39H32O14	725	0.79
5	10.155	ent-Epicatechin(4*α*- > 8)catechin	C30H26O12	579	0.60
6	10.434	Ent-epicatechin-(2*α*- > 7,4*α*- > 8)-epicatechin 3-galactoside	C36H34O17	739	1.38
7	10.898	Ent-epicatechin-(2*α*- > 7,4*α*- > 8)-epicatechin 3-galactoside	C36H34O17	739	1.34
8	11.213	3-*O*-*β*-D-Galactopyranosylproanthocyanidin A5'	C36H34O17	739	1.34
9	11.877	2-oxalobenzoic acid	C9H6O5	195	0.66
10	12.031	Prenyl caffeate	C14H16O4	249	0.95
11	12.143	Prenyl caffeate	C14H16O4	249	2.38
12	12.653	5,7,3′,5′-Tetrahydroxyflavanone	C15H12O7	305	3.61
13	12.898	5,7,3′,5′-Tetrahydroxyflavanone	C15H12O7	305	7.88
14	13.39	Quercetin	C15H10O7	303	16.22
15	13.918	Fustin	C15H12O6	289	3.16
16	14.339	Eriodictyol	C15H12O6	289	2.61
17	16.717	Fisetin	C15H10O6	287	1.84
18	17.295	C16 Spinganine	C16H35NO2	274	27.23
19	17.588	Hexosylsphingosine	C24H47NO8	478	0.56
20	18.661	Dehydrophytospingosine	C18H37NO3	316	0.34
21	19.356	Phytospingosine	C18H39NO3	318	1.16
22	20.643	Lauroyl diethanolamide	C16H33NO3	288	0.78
23	21.621	Coniferyl benzoate	C17H16O4	285	1.70
24	22.378	2',6'-Dihydroxy-4'-methoxy-3'-methyldihydrochalcone	C17H18O4	287	0.31
25	33.356	Tridodecylamine	C36H75N	522	1.62
26	34.975	dimethyldistearylamine	C38H79N	551	0.33

### Biological activity probability

The PASS server disclosed the biological activity prediction of BC. Surprisingly, only three of the seven evaluated bioactive substances were associated with antidiabetic activity via AG inhibition (Table 4). The mechanism of astilbin, neoastilbin, and quercetin 3-rhamnoside exhibited the highest possibility (Pa > 0.7) as AG inhibitors, among others. The possibility that the other four substances, 5,7,3′,5′-tetrahydroxyflavanone, fisetin, quercetin, and C16 spinganine, are effective AG inhibitors is low. The global development of therapeutic candidates for the treatment of several disorders, including T2DM, remains difficult. Today, the fast growth of computer science, which has resulted in a computational method, has inspired a great deal of interest in the preclinical evaluation of several drug candidates. In addition, it may lower costs and decrease failure rates in the clinical phase of drug research [36]. The present investigation predicted that methyl *β*-D-glucopyranoside, astilbin, neoastilbin, and quercetin 3-rhamnoside from BC might be good antidiabetic candidates.

**Table 4:** Predicted Pa and Pi values of phytochemical BC for AG inhibition using PASS online server

No	Compounds	Pa	Pi	Peak area (%)
1	Methyl *β-*D-glucopyranoside	0.765	0.001	8.98
2	Astilbin	0.746	0.001	10.49
3	Neoastilbin	0.746	0.001	14.10
4	Quercetin 3-rhamnoside	0,736	0.001	14.10
5	5,7,3′,5′-Tetrahydroxyflavanone	0,196	0,057	5.35
6	Fisetin	0,222	0,004	6.05
7	Quercetin	0,166	0,079	16.22
8	C16 Spinganine	0.008	0.005	27.23

### Molecular interaction and 2D/3D chemical visualization

**Fig. 1:**
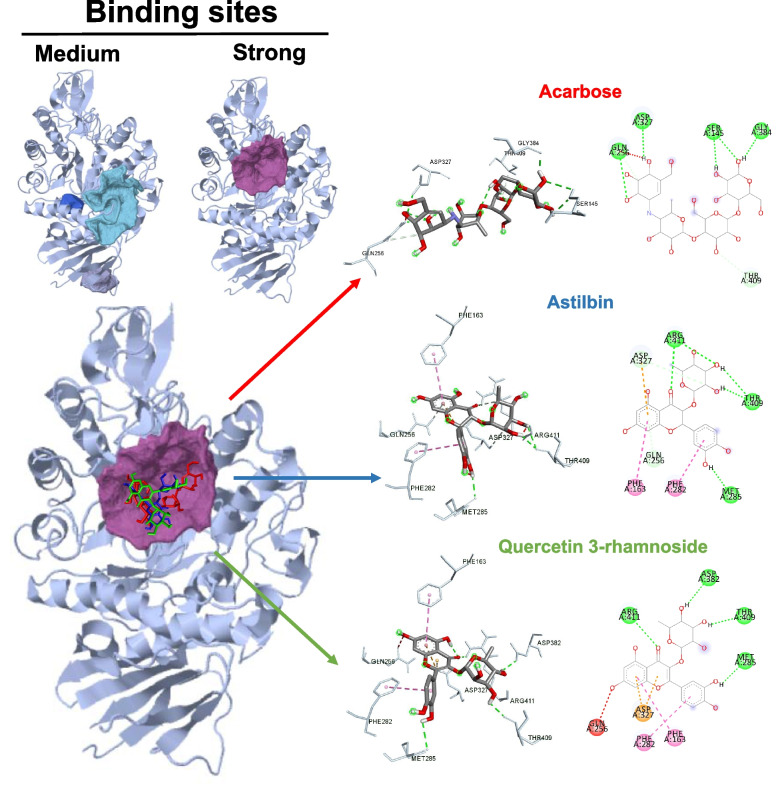
2D and 3D Analysis through Discovery Studio: Alpha glucosidase pocket binding sites for active compounds of *Bauhinia strychnifolia* Craib and acarbose (positive control)

The molecular docking study on the high possibility of BC compounds and acarbose (positive control) demonstrated that the best poses of astilbin and quercetin 3-rhamnoside showed lower binding energy than acarbose in all programs (Table 5), indicating that both compounds will have the strongest binding affinity to the enzyme; AG may therefore exhibit the strongest enzyme inhibition [37]. This indicates that astilbin and quercetin 3-rhamnoside should be developed as AG inhibitors. In a comprehensive investigation of the binding of the top docked poses of astilbin, quercetin 3-rhamnoside, and acarbose at the same pocket binding site, the top docked poses of astilbin, quercetin 3-rhamnoside, and acarbose are shown in Fig. 1. Astilbin had a total of seven hydrogen bond interactions, but quercetin 3-rhamnoside exhibited just four. Although acarbose may have a strong seven-hydrogen interaction with AG, astilbin exhibited the highest affinity for the target, surpassing both acarbose and quercetin 3-rhamnoside, because astilbin exhibited not only the seven-hydrogen interaction but also the electrostatic (orange) and alkyl/pi-alkyl interactions (pink). Overall, the molecular docking investigations shed light on the ability of astilbin to inhibit AG and on prospective methodologies for structure-based design or modification of astilbin with enhanced inhibitory action against AG.

**Table 5:** Molecular docking results and inhibition constant values of AG inhibitory activity of BC

No	Ligand	Binding energy (kcal/mol)			Anti-diabetes Activity (inhibition constant)
		Arguslab	Autodock Vina	Autodock	
1	Acarbose (Positive control)	-7.58662	-7.8	-5.17	161.07 µM
2	Methyl *β*-D-glucopyranoside	-7.24823	-6.0	-2.42	16.86 mM
3	Astilbin	-8.47852	-9.4	-7.54	2.96 µM
4	Quercetin 3-rhamnoside	-8.64996	-9.2	-7.68	2.34 µM

### Molecular dynamics

**Fig. 2:**
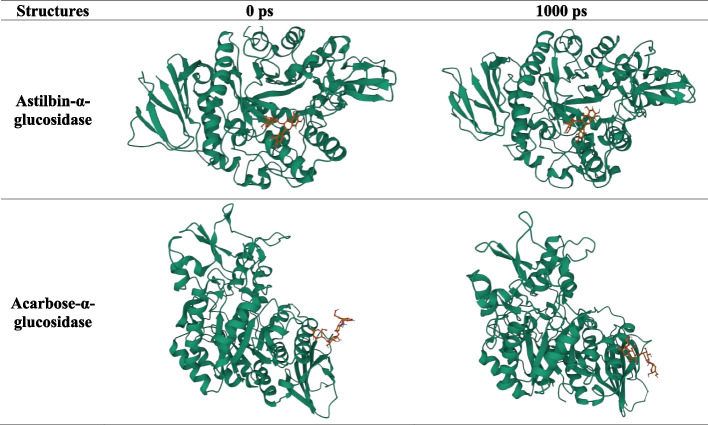
Initial and final structures of compound-alpha glucosidase complexes during the molecular dynamics

MD simulation is used to examine the ligand-enzyme binding details across time [38]. The structure of the receptor-ligand complex with the lowest docking score used as the starting geometry for MD simulation. The beginning and final pictures of all AG-inhibitor complexes are shown in Fig. 2: Snapshots of AG-inhibitors complexes revealed that the complexes underwent minimal modifications during MD simulation period.

**Fig. 3:**
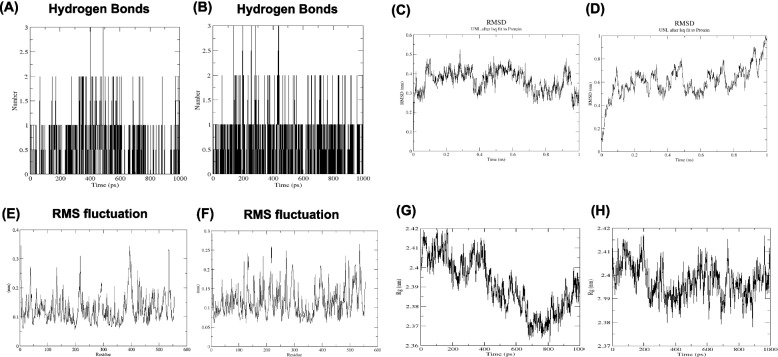
Hydrogen bonding of AG with (A) astilbin and (B) acarbose. Root mean square deviation (RMSD) of AG with (C) astilbin and (D) acarbose. Root mean square fluctuation (RMSF) values of AG with (E) astilbin and (F) acarbose. Radius of gyration Rg (Å) values of AG with (G) astilbin and (H) acarbose

Some variables, including RMSF, RMSD, and Rg, indicate a superior binding pattern. RMSD analysis contributes to the conformational stability of each complex [39]. Therefore, RMSD analyses were conducted on AG in the presence of inhibitors. According to Fig. 3 (A) and (B) the number of hydrogen bonds fluctuates between 0 and 3 for Astilbin, 0 same as Acarbose (positive control). According to these results, the main hydrogen bonds found in the best docked pose were maintained during the MD simulation. As it could be seen in Fig. 3 (C) and (D), The maximum RMSD value for complexes of AG with acarbose and astilbin were1 nm, and 0.55 nm, respectively. The RMSD value of astilbin was less than acarbose (positive control) during the simulated complexes indicating the stability of astilbin with AG complex in the period of the simulation time. This sounds reasonable regarding the fact that astilbin binds AG with stability. RMSF provides details on the flexibility of residues in the presence of inhibitors. The RMSF parameter of the acarbose-AG and astilbin-AG complex is depicted in Figs. 3E and F. The acarbose-AG combination was discovered to have a lower fluctuation pattern than the astilbin-AG complex. This implies that the presence of an astilbin inhibitor has a greater impact on the flexibility of the residue during simulations than acarbose. To examine the compactness of digestive enzymes in the presence of inhibitors, the Rg parameter was determined. As shown in Fig. 3G and H, the Rg parameter of acarbose-AG varied near 2.38 nm and developed to a maximum of 2.42 nm, whereas the Rg parameter of astilbin-AG complex fluctuated near 2.36 nm and increased to a maximum of 2.42 nm. Astibilbin-AG revealed a lower Rg value than acarbose-AG, indicating fewer conformational changes during simulation. As seen in Figs. 3G and H, the astilbin-AG complex exhibited a lower Rg than the acarbose-AG complex, indicating fewer conformational changes during simulation. The greater the Rg, the less compact the complex was, and vice versa. From our simulation outputs, it shows that AG in the presence of astilbin exhibits more compactness than acarbose.

### Solvent consideration

**Fig. 4:**
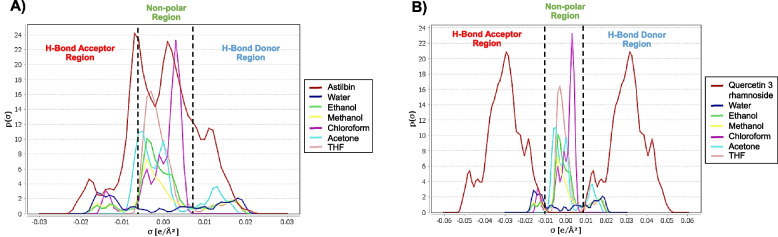
The region of − 0.008 eA − 2 < σ <  + 0.008 eA − 2 indicates London dispersion forces in heptane. Peaks at σhb >  + 0.008eA − 2 indicate hydrogen bonding energy and the presence of hydrogen bond donor, while peaks at σhb <  + 0.008eA − 2 indicate the presence of a hydrogen bond acceptor group. A higher negative value of µ(σ) indicates an increasing interaction between molecules, while a higher positive value indicates an increase in repellant behavior

The surface charge density is a useful molecular descriptor for determining the polarity of molecular surfaces. In statistical thermodynamics, the intermolecular forces may be quantified using the surface charge density. Quantum chemical information of polarization charge density is crucial for the investigation of molecular interactions [40]. To investigate the interaction between solute and solvent molecules, the sigma profile of molecules was examined and generated. This profile provided the relative surface polarity of molecules, as shown in Fig. 4. The sigma profile depicts the probability distribution of the surface charge density around molecules [41]. Figure 4 depicts the polarization charge densities of water; ethanol; methanol; chloroform; acetone; THF; and the two best predicted compounds, astilbin and quercetin 3-rhamnoside, with the area representing the sigma profiles of the respective molecules. As indicated in Table 6, COSMOquick was used to determine the sigma profile of molecules, after which the solubility properties of molecules in the solvent was analyzed. Hydrogen bond donor, nonpolar, and hydrogen bond acceptor regions make up the sigma profile [42]. The range between 0.008 e/A^2^ and + 0.008 e/A^2^ represents London dispersion forces. Hydrogen bonding energy and the presence of a hydrogen bond donor group are indicated by peaks at sigma >  + 0.008 e/A2, while peaks at sigma <  + 0.008 e/A2 indicate the existence of a hydrogen bond acceptor group. A greater negative number suggests a rise in attracting behavior, whereas a greater positive value indicates a rise in repellent conduct [43]. Astilbin was primarily located in the nonpolar area, indicating that the molecule was successfully extracted using insoluble solvents. As indicated in Table 6, astilbin has the highest solubility in tetrahydrofuran after calculations with COSMOquick. In contrast, quercetin 3-rhamnoside dominated the polar region, primarily as a hydrogen donor and hydrogen acceptor. After COSMOQuick examination, the solvent employed for extraction was highly soluble in ethanol, methanol, acetone, and THF.

**Table 6:** Predict the solubility of solvents in the active components of BC using COSMOquick

Solute	Solvent	Dielectric constant	Solubility (g/L)
Astilbin	Water	78.5	4 × 10^–3^
	Ethanol	24.3	1.22 × 10^2^
	Methanol	32.6	3.06 × 10^2^
	Chloroform	4.7	3.0 × 10^–4^
	Acetone	20.7	4.53 × 10^2^
	Tetrahydrofuran	7.4	1.43 × 10^3^
	Ethyl acetate	6.02	5.0
	Hexane	1.9	3.36 × 10^–10^
Quercetin 3-rhamnoside	Water	78.5	1.04 × 10^–1^
	Ethanol	24.3	1.38 × 10^3^
	Methanol	32.6	1.43 × 10^3^
	Chloroform	4.7	7.0 × 10^–5^
	Acetone	20.7	1.43 × 10^3^
	Tetrahydrofuran	7.4	1.43 × 10^3^
	Ethyl acetate	6.02	61.2
	Hexane	1.9	4.10 × 10^–10^

### QSAR analysis

QSAR analysis is a method used to relate the physicochemical features of a variety of chemical compounds with AG inhibition. Using EasyQSAR 1.0, the 2D-QSAR study was implemented to confirm and associate the identified AG inhibitory findings with the structural properties of the tested compounds. Molecular descriptors from SwissADME that correlate chemical structures with biological activity were represented as IC_50_ in terms of µg mL-1 in Eq. 4. The IC_50_ value of astilbin was calculated from the molecular descriptor, and values were added for calculation in Eq. 2. The predicted IC_50_ of astilbin was 22.92 µg mL-1.

4 $$\mathrm{PredictedIC}50=-320+608(\mathrm{HA})-112(\mathrm{HD})-28(\mathrm{TPSA})+400(\mathrm{LogP})$$

### Quantum compound computations

**Fig. 5:**
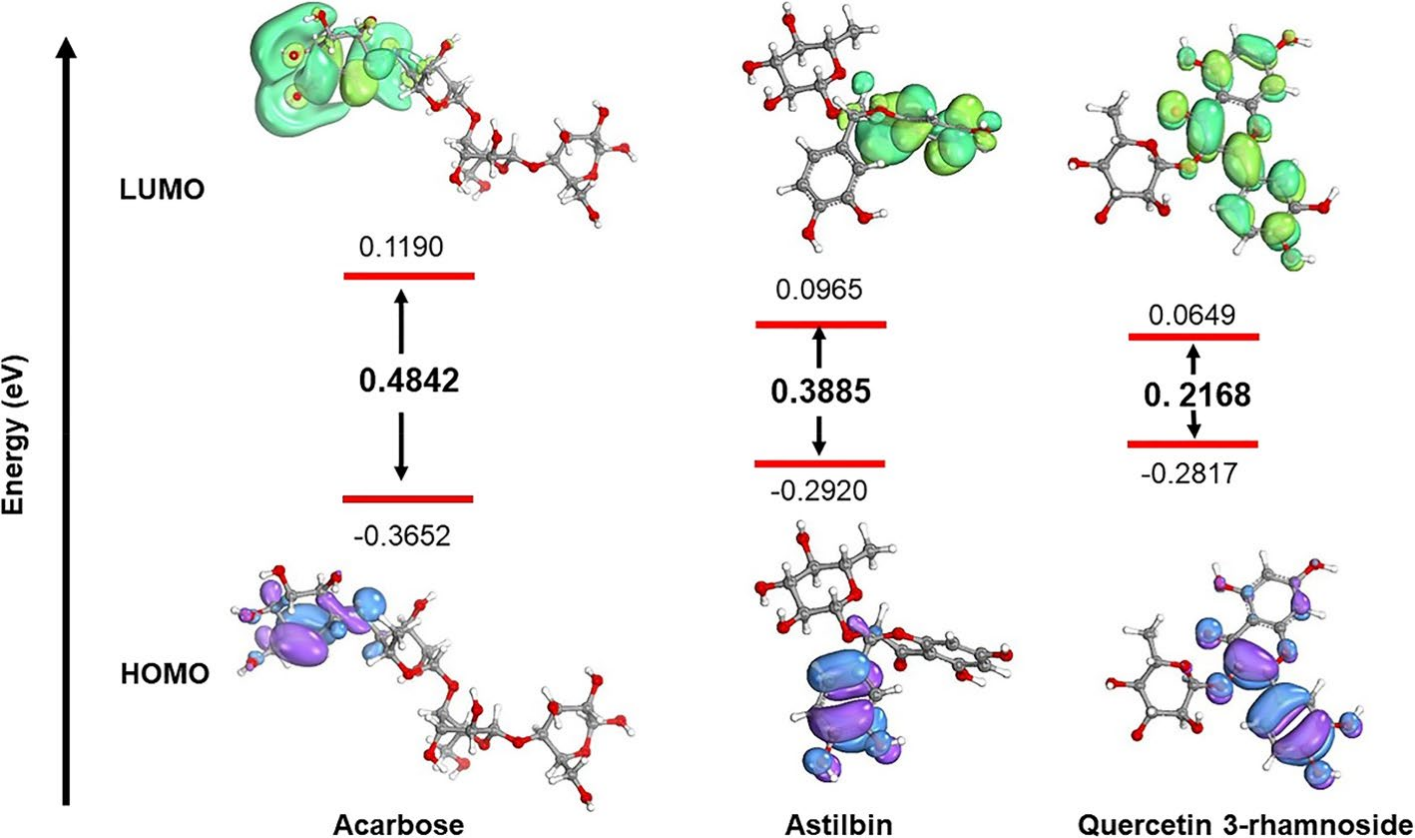
The HOMO–LUMO energy gap of acarbose (positive), astilbin, and quercetin 3-rhamnoside

The difference in energy between HOMO and LUMO is correlated to the chemical stability of molecules [44]. Less stable molecules possess a small HOMO–LUMO energy gap, while more stable compounds possess a large HOMO–LUMO energy gap [45]. The overall energy gap between HOMO and LUMO is depicted in Fig. 5. The HOMO–LUMO energy gap value of quercetin 3-rhamnoside (0.2168 eV) is less than that of astilbin (0.3885 eV). Consequently, astilbin is more stable than quercetin 3-rhamnoside due to its large HOMO–LUMO energy gap.

### NMR characterization

The compound astilbin was isolated as a white solid (102 mg). The molecular formula of C_21_H_22_O_11_ was established by low-resolution EI-MS [*m/z*], EI-MS: *m/z* (%), 413.8 (5), 332 (7), 303.8 (35), 274.8 (45), 258.8 (15), 176.9 (20), 152.9 (100), 132.9 (70), 122.9 (50), 119.0 (35), 107.0 (20), 77.9 (100). IR spectrum absorption bands for the hydroxyl group at 3401 cm^−1^, aromatic C = C stretching at 1524 cm^−1^ and ketone C = O stretching at 1604. The UV spectrum showed absorption bands at 207 and 290 nm.

**Fig. 6:**
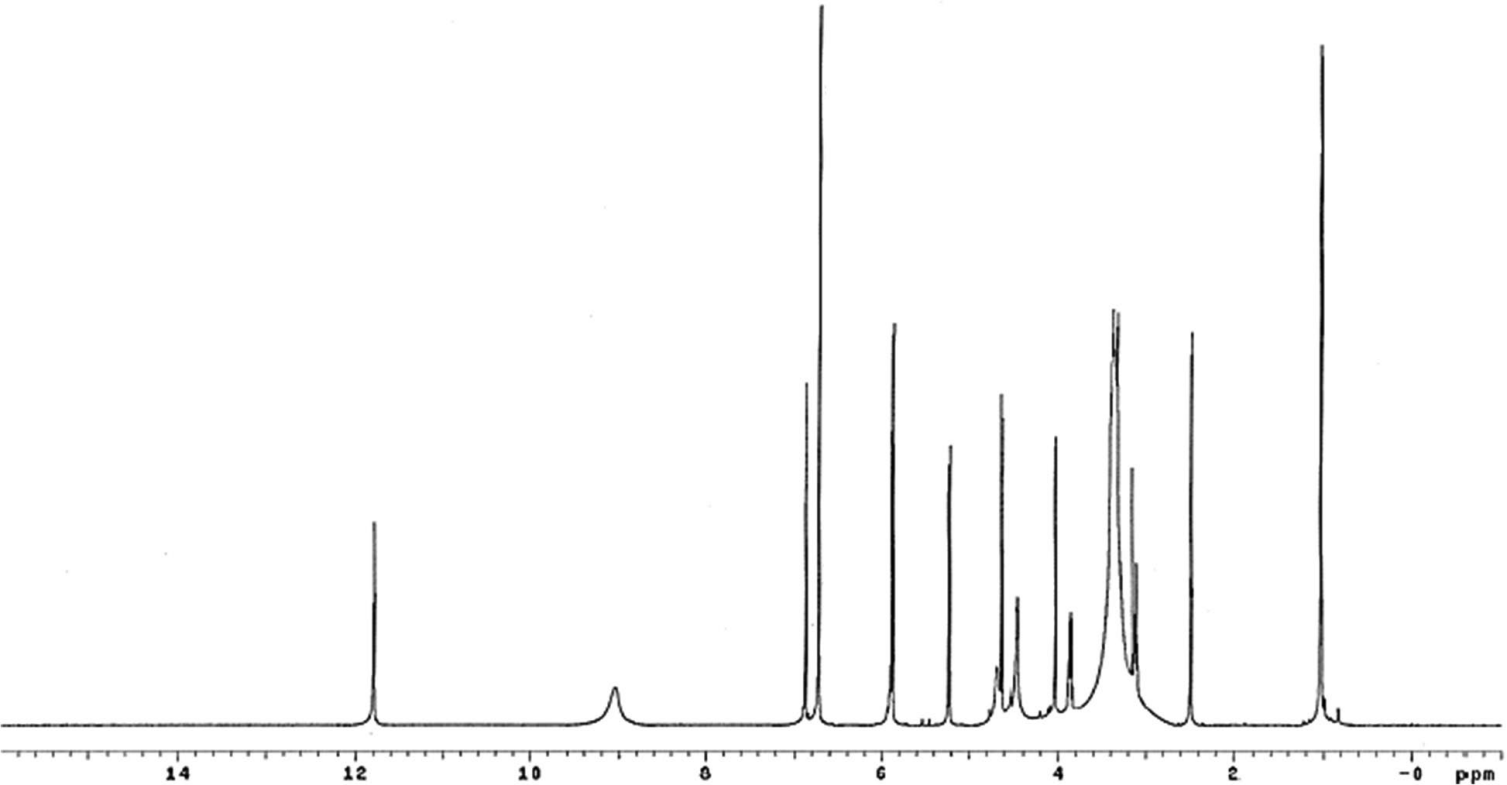
^1^H NMR spectrum of astilbin (DMSO-d_6_; 500 MHz)

The ^1^H-NMR spectrum recorded in 500 MHz, DMSO-*d*_*6*_ exhibited signals to ring B in the flavanonol structure by means of a distinctive coupling pattern at *δ* 4.64 (1H, *d*, *J* = 10, H-3), 5.24 (1H, *d*, *J* = 10.0 Hz, H-2), five aromatic signals at *δ* 5.87 (1H, *d*, *J* = 2.2 Hz, H-8), 5.90 (1H, *d*, *J* = 2.3 Hz, H-6), 6.73 (2H, *d*, *J* = 1.0 Hz, H-5′, 6′), *δ* 6.88 (1H, *br, s*, H-2′), and one anomeric proton signal at *δ* 4.04 (1H, *d*, *J* = 1.0 Hz, H-1′′). The three methine protons in the sugar molecule were represented as multiplet signals at *δ* 3.88 (1H, *m*, *J* = 6.2, 9.5 Hz, H-5′′), *δ* 3.39 (2H, *m*, H-2′′, 3′′), *δ* 3.13 (1H, *t*, *J* = 9.3 Hz, H-4′′) and one methyl proton at *δ* 1.04 (3H, *d*, *J* = 6.2 Hz, H-6′′). Four phenolic hydroxyl groups at *δ* 4.48 (1H, *br, s*, OH), 4.68 (1H, *br, s*, OH), 9.04, (1H, *br, s*, OH) and 11.79 (1H, *s*, OH) are shown in Fig. 6.

**Fig. 7:**
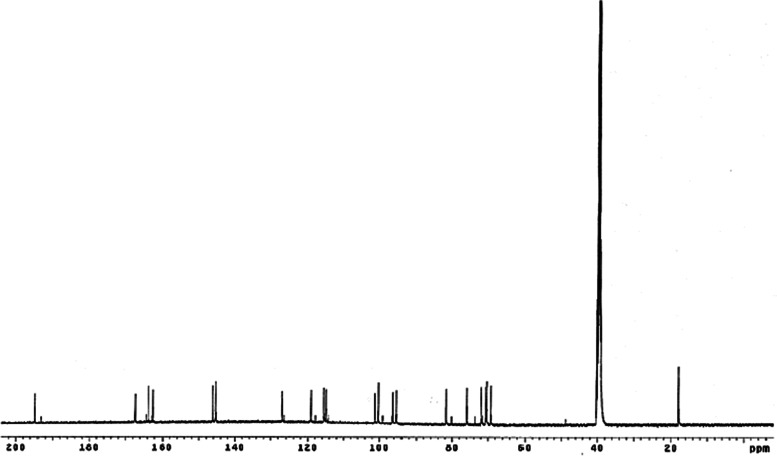
^13^C NMR spectrum of astilbin (DMSO-d_6_; 500 MHz)

The ^13^C NMR spectrum recorded at 500 MHz in DMSO-*d*_*6*_ exhibited signals corresponding to ring B in the flavanonol molecule (*δ* 75.8, 81.7), five aromatic methine signals (*δ* 95.2, 96.2, 114.9, 115.5, 119.0), three quaternary carbons (*δ* 162.3, 101.2, 127.1), signals characteristic of phenolic carbons (163.6, 167.2, 146.1, 145.3) and one ketone (*δ* 194.6). The sugar unit showed one anomeric carbon at *δ* 100.2, four methine carbons (*δ* 69.1, 70.3, 70.6, 71.8) and one methyl carbon (*δ* 17.9), as shown in Fig. 7. Therefore, the spectroscopic data were compared with a previous report [46].

### AG inhibition test

**Fig. 8:**
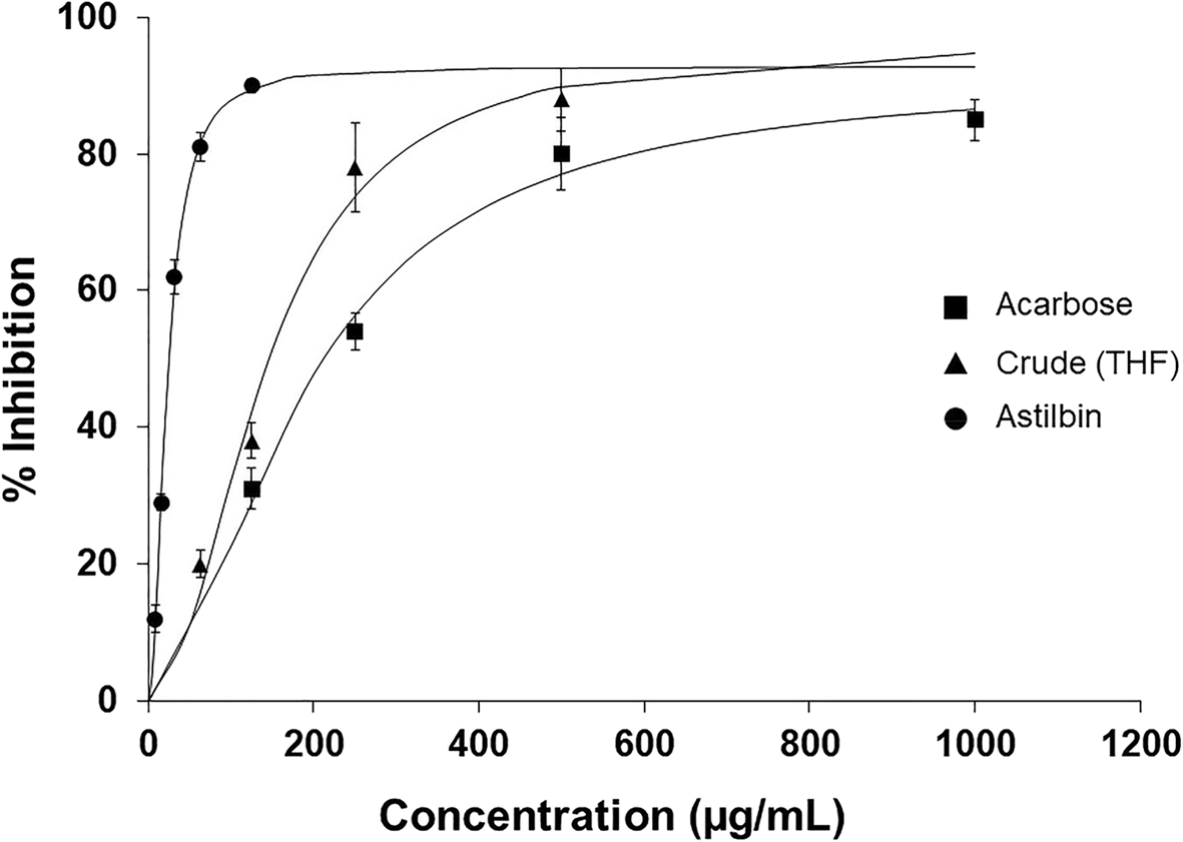
Inhibition of α-glucosidase enzyme by acarbose, crude compounds extracted with THF, and astilbin

Acarbose, a typical medicine for the treatment of diabetes, inhibits AG. Acarbose has an IC_50_ of 190 ± 6.97 µg mL-1. The THF BC extract inhibited AG at a concentration of 158 ± 1.30 µg mL-1. According to COSMOquick's solvent estimate, astilbin and quercetin 3-rhamnoside were the most abundant chemicals in BC following THF extraction. Quercetin 3-rhamnoside has been shown to significantly inhibit AG in a previous study [47], while astilbin extracted from BC stems was undertaken for its AG inhibitory efficacy to confirm its ability to inhibit AG. Therefore, astilbin was isolated further, and other chemicals were excluded from AG inhibition testing in this investigation. The IC_50_ of astilbin against AG was determined to be 22.51 ± 0.70 µg mL-1 as shown in Fig. 8.

## Conclusion

Phytochemical analysis, AG inhibition, and docking investigations of pure astilbin from BC stems were undertaken in this paper. In comparison to the commercial medication acarbose, column-purified fraction 5 of BC inhibits AG to a greater degree. Comparable to past research, 'fraction-5' has a strong AG inhibition, and phytochemical analysis verified the presence of astilbin. AG activity was strongly inhibited by the THF BC extract and, in particular, astilbin, as shown by the inhibition kinetics research. The greater IC_50_ of astilbin (22.51 ± 0.70 µg mL-1) compared to that of acarbose (190 ± 6.97 µg mL-1) shows that the compounds found in 'fraction 5' might be efficient AG inhibitors for the control of T2DM. LC–MS analysis of pure 'fraction 5' led to the discovery of a chemical (astilbin), among which quercetin 3-rhamnoside was previously recognized for its AG inhibition activity. The docking analysis also revealed that astilbin and quercetin 3-rhamnoside had a greater binding energy than the well-known medication acarbose against AG, indicating that the found compounds are more effective AG inhibitors than acarbose. Astilbin's potent AG inhibitory activity shows potential for its usage as a treatment for T2DM. Despite the fact that the in vitro investigation reveals outstanding AG inhibitory activity, in vivo studies are necessary to determine the therapeutic potential of these molecules before they can be considered for the treatment of T2DM.

## Data Availability

All data generated or analysed during this study are included in this published article.

## Abbreviations

THF: Tetrahydrofuran
BC: *Bauhinia strychnifolia* Craib
AG: Alpha-glucosidase
QSAR: Quantitative structure–activity relationship
PASS: Prediction of activity spectra for substances
LC–MS/MS: Liquid Chromatography with tandem mass spectrometry
NMR: Nuclear magnetic resonance
T2DM: Type 2 diabetes
IC_50_: The half-maximal inhibitory concentration
